# Proteolytic shedding of the prion protein: Uncovering “new” biological implications of a conserved cleavage event

**DOI:** 10.4103/NRR.NRR-D-25-00013

**Published:** 2025-06-19

**Authors:** Feizhi Song, Valerija Kovac, Behnam Mohammadi, Josephine E. Pippi, Vladka Curin Serbec, Markus Glatzel, Hermann C. Altmeppen

**Affiliations:** Institute of Neuropathology, University Medical Center Hamburg-Eppendorf (UKE), Hamburg, Germany; Centre for Immunology and Development, Slovenian Institute for Transfusion Medicine, Ljubljana, Slovenia

Novel insights into complex biological processes very often critically depend on the establishment of new potent read-out tools and improved protocols. A lot has been learned over the past four decades on physiological functions and, importantly, disease-related roles of the prion protein (PrP), a relatively broadly expressed membrane-anchored glycoprotein with high levels in several cell types of the nervous and immune system and with well-established key roles in different progressive and fatal neurodegenerative protein misfolding diseases (proteopathies). However, while several controversies and unclarities remain even for these widely accepted involvements, currently unexplored (and unexpected) facets and functions may still wait to be discovered. New light might be shed into these aspects by a better understanding of potential intrinsic roles of previously largely unconsidered post-translationally generated forms or fragments of PrP, for instance those resulting from endogenous proteolytic cleavage (Mohammadi et al., 2022; Vanni et al., 2022). In fact, membrane-bound full-length (FL) PrP, the form most research of the past has focused on, may not even represent the majority of total PrP in the brain (Vanni et al., 2022). A nearly FL form released from cells by a constitutive and very membrane-proximate proteolytic cleavage event (“shedding”) makes up for a rather small yet relevant fraction and is of emerging interest. This physiological, anchorless, and predominantly double-glycosylated form, now called “shed PrP” (sPrP), has repeatedly been reported in the past (e.g., Parizek et al., 2001), for instance, in cell culture media supernatants or body fluids, yet its mechanistic origin and biological relevance remained obscure for a long time. The latter, to a great deal, is due to technical challenges differentiating this fragment from excess FL-PrP present in most biological specimens (e.g., tissue homogenates; but even in body fluids or cell culture supernatants, FL-PrP is present on cellular membrane debris and physiologically released extracellular vesicles (EVs)). Both forms are of similar molecular weight and share structure and sequence and, hence, epitopes for most available antibodies used for detection in standard laboratory techniques (Mohammadi et al., 2022). Besides recently improved protocols to differentiate and quantify the abundance of different PrP “proteoforms” by immunoblotting (Vanni et al., 2022), cleavage site-directed antibodies previously presented for the reliable detection of rodent sPrP have become a convenient tool to systematically, highly specifically, and comparably comfortably assess sPrP with various methods, as these antibodies are “blind” for the just few amino acids (plus the C-terminally attached glycosylphosphatidylinositol anchor) longer FL form (Linsenmeier et al., 2021; Mohammadi et al., 2022). However, given that many key aspects regarding PrP shedding, such as cleavage site and responsible protease, remained uncharacterized for the human body, our groups recently set out to unravel many unknowns in this regard. We eventually succeeded in identifying the shedding site (Y226↓Q227) in human PrP and recently presented an in-depth characterization of respective antibodies exclusively detecting sPrP (Song et al., 2024). As expected from previous mouse data, we revealed that the shedding of human PrP is likewise strictly dependent on the metalloprotease ADAM10, and we did not find any evidence for alternative proteolytic cleavages in the vicinity of Y226 (Song et al., 2024). But what makes us confident that PrP shedding is a relevant and understudied process, and sPrP a fragment of potentially broader biomedical interest? Why do we think there are still a lot of unknowns regarding this aspect that urgently need to be unraveled?

**Figure 1:**
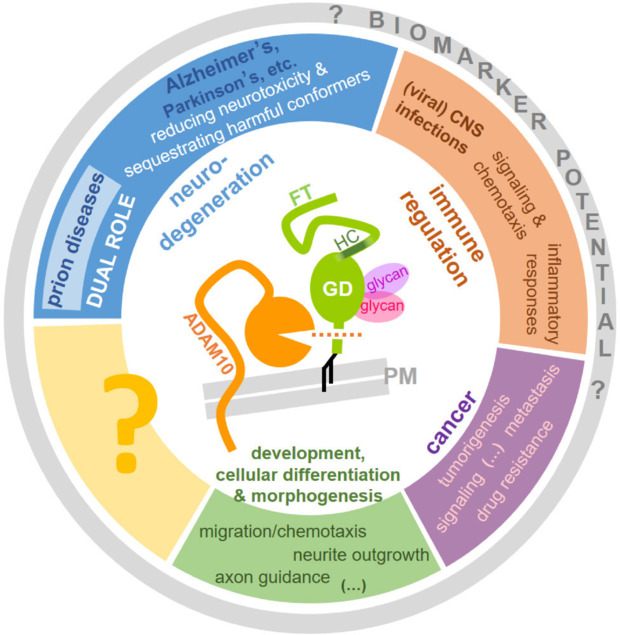
Scheme summarizing confirmed as well as potential/suggested roles of prion protein (PrP) shedding in diverse biological processes. The prion protein (green) is attached to the plasma membrane (PM) via a glycosylphosphatidylinositol anchor (black) and contains up to two N-glycans (pink) attached to the structured C-terminal half of the protein (GD: globular domain). A hydrophobic core (HC) sequence links the GD and the N-terminal intrinsically disordered flexible tail (FT). Membrane proximate cleavage (dotted orange line) by ADAM10 (orange) releases PrP into the extracellular space. This process and the resulting shed PrP (sPrP) appear to have relevance in a variety of (patho)physiological processes, ranging from homeostatic functions to neurodegenerative diseases, inflammation, and cancer, as indicated here and discussed in the main text. Specific detection of sPrP in body fluids may hold potential as a disease- or therapy-relevant biomarker in these fields (grey circle) and new biological implications may emerge in the future (yellow box/question mark). Detailed studies in these directions will profit from newly established research tools, such as cleavage site-specific antibodies for the detection of sPrP.

In several neurodegenerative proteopathies (**Figure 1**, blue area), cell surface PrP acts as a toxicity receptor/mediator for harmful extracellular disease-characteristic protein assemblies. Fittingly, ADAM10-mediated shedding could be of therapeutic interest, as it reduces PrP membrane levels and, hence, toxic effects of Alzheimer’s disease-associated Aβ oligomers in respective *in vitro* models (Jarosz-Griffiths et al., 2019). Moreover, using our sPrP-specific antibodies, we revealed that, in the brains of Alzheimer’s disease patients and mouse models for amyloidosis, sPrP colocalizes with a fraction of extracellular plaques, indicative of a binding and sequestrating activity towards harmful diffusible protein/peptide assemblies (Linsenmeier et al., 2021; Mohammadi et al., 2022; Song et al., 2024). The latter feature was also observed in brains affected by human (e.g., Creutzfeldt-Jakob disease) and animal (e.g., bovine spongiform encephalopathy in cattle) prion diseases (PrD), in which progressive templated misfolding and aggregation of PrP itself represents the key pathogenic event (Lukan et al., 2014; Linsenmeier et al., 2021; Song et al., 2024). In a prion disease mouse model, sPrP levels appeared to inversely correlate with the formation of the misfolded isoform (PrP^Sc^) and conditional depletion of neuronal ADAM10 in the forebrain caused elevated neuropathology and a significantly shortened period to reach terminal disease (Altmeppen et al., 2015; Mohammadi et al., 2022). While these findings suggest beneficial effects of PrP shedding in neurodegeneration, a potential dual role in PrD also needs to be considered: shedding, to some extent, might be involved in the production of anchorless, diffusible assemblies of PrP^Sc^, which may even represent bona fide “prions” (i.e., the infectious units or “seeds” characteristic for PrD) and could be involved in the dissemination of pathology within the brain (and body) and –conceivably– even in the transmission between individuals (Lukan et al., 2014; Altmeppen et al., 2015; Song et al., 2024). Our sPrP-specific antibodies will support future studies not only in humans (Song et al., 2024) and laboratory rodents (Linsenmeier et al., 2021), but also in some mammalian species affected by PrD, such as cattle, sheep, and deer (Song et al., 2024). Given that the latter two are naturally affected by the most contagious forms of mammalian prion diseases (i.e., Scrapie and chronic wasting disease, respectively), with horizontal transmission occurring frequently and efficiently, it will be of particular interest to study the potential influence of the ADAM10-mediated PrP shedding in prion infectivity in body fluids and excrement released into the environment. Moreover, recent technical advances allowing for high-resolution structural elucidation of prion fibrils, possibly combined with the use of our cleavage site-directed antibodies, may reveal if and to which extent sPrP in a misfolded conformation is incorporated into these structures and how the interaction of prion aggregates and bona fide sPrP presents at the molecular level. Such insight could foster our understanding of prion strains and strain-typical neuropathological and clinical presentations among various prion disease subtypes.

Details on the aforementioned aspects related to PrP shedding in neurodegeneration can be found in the referenced articles, yet completely new topics related to sPrP are currently emerging and certainly worth investigating as well (**Figure 1**, differently colored areas). Compared to shorter N-terminal PrP fragments released by cells, sPrP is relatively stable and would qualify as a ligand in intercellular communication. Release of PrP has been reported for platelets (Perini et al., 1996), lymphoid tissue and splenocytes (Parizek et al., 2001), and mast cells (Haddon et al., 2009; Willows et al., 2024), suggesting involvement in immune responses. Fittingly, the ADAM10-mediated shedding has been linked with (rather detrimental roles) in inflammatory processes and immune regulation of viral brain infections, for instance by acting as a ligand in a communication cascade between different cell types eventually causing excessive and harmful monocyte recruitment to the brain in HIV-associated neuropathogenesis (Megra et al., 2017). In another recent study, a reactive increase in PrP shedding was linked with a decreased control of perinatal cytomegalovirus infection, with sPrP binding to and attenuating CD8 T cell responses (Karner et al., 2024). However, rather beneficial anti-inflammatory roles of released PrP, for instance by reducing bacterial lipopolysaccharide-induced toxicity pathways, have also been reported (Mantuano et al., 2022), thus highlighting the need for a more detailed and differentiated picture of the ADAM10-mediated PrP shedding in these complex immunoregulatory aspects.

In addition, suggested roles for PrP in development, differentiation, and morphogenesis (including processes like the epithelial-to-mesenchymal transition (Lailler et al., 2024)) as well as tissue homeostasis may be linked with sPrP. The latter may also be the physiological ligand involved in maintaining proper myelination of peripheral axons by Schwann cells (Kuffer et al., 2016) and in the chemotactic outgrowth of neurites and cellular migration (Amin et al., 2016; Mantuano et al., 2020).

Inflammatory processes and epithelial-to-mesenchymal transition are also relevant aspects of tumorigenesis and cancer progression (Lailler et al., 2024). Moreover, it has been reported for several cancer types that increased ADAM10 expression/activity as well as elevated PrP levels are indicators of a poor prognosis, which suggests harmful mechanistic involvement of both players. Although both molecules have so far been discussed without a direct connection in the cancer context, it appears plausible that the “product” of protease (here: ADAM10) and substrate (here: PrP), i.e., sPrP, is somehow mechanistically involved or may at least serve as a clinically traceable biomarker. First reports with regard to PrP and cancer already suggested that sPrP might be a ligand in auto- or paracrine signaling events during development and cellular proliferation of certain tumors (Provenzano et al., 2017), while another study revealed that sPrP directly binds to and sequesters certain chemotherapeutics and might thereby contribute to drug resistance in certain cancer types (Wiegmans et al., 2019). These and other studies point towards clinically relevant roles of PrP in cancer, which might in part be orchestrated by PrP shedding. Further studies enabling new insight into these directions are clearly warranted.

While the aforementioned studies nicely paved the way for linking sPrP with (patho)physiological processes beyond neurodegeneration, we have recently recommended careful interpretation of certain findings, as in many of these studies the authors actually either used recombinant PrP (expecting this would mimick sPrP) and/or did not strictly discriminate between sPrP on the one hand and PrP located at the surface of EVs on the other hand (Mohammadi et al., 2022). Again, a more differentiated view and detailed insight could likely be gained by using sPrP-specific antibodies. A fraction of sPrP, after release, secondarily sticks back to cell surfaces or EVs (Song et al., 2024). Whether this is due to specific interactions with surface receptors (possibly causing downstream effects) or rather random binding to membranes still needs to be elucidated, but it clearly has technical implications: for instance, ultracentrifugation of biological samples (with the intention to separate sPrP from EVs) would also pull down a large part of sPrP molecules being attached to the corona of EVs (Song et al., 2024). Thus, to get the true picture on total amounts of sPrP in a given biological sample, be it extracellular, attached to cells and EVs, or even taken up in endosomal compartments en route to lysosomal degradation, the use of cleavage site-directed antibodies currently seems to be the most comfortable and reliable way.

*The authors would like to apologize to all colleagues whose important contributions could not be referenced due to space limitations.*

*This work was supported by the CJD Foundation, USA, the Alzheimer Forschung Initiative (AFI) e.V., Germany, and Werner-Otto-Stiftung, Germany (all to HCA), China Scholarship Council (grant #202108080249 to FS), Deutsche Forschungsgemeinschaft (DFG) CRC877 “Proteolysis as a regulatory event in pathophysiology” (project A12 to MG), Slovene Research and Innovation Agency (grant number P4-0176 to VCS).*
